# Therapeutic potential of antiviral drugs targeting chemorefractory colorectal adenocarcinoma cells overexpressing endogenous retroviral elements

**DOI:** 10.1186/s13046-015-0199-5

**Published:** 2015-08-12

**Authors:** David Díaz-Carballo, Ali Haydar Acikelli, Jacqueline Klein, Holger Jastrow, Philipp Dammann, Thomas Wyganowski, Cihan Guemues, Sebastian Gustmann, Walter Bardenheuer, Sascha Malak, Nora Sophia Tefett, Veria Khosrawipour, Urs Giger-Pabst, Andrea Tannapfel, Dirk Strumberg

**Affiliations:** Institute for Molecular Oncology and Experimental Therapeutics, Division of Oncology and Hematology, Marienhospital Herne, Ruhr University of Bochum, Medical School, Marienhospital Herne, Duengelstr. 33, 44623 Herne, Germany; Institute of Anatomy and Experimental Morphology, University of Duisburg-Essen, Medical School, Essen, Germany; Central Animal Laboratory, University of Duisburg-Essen, Medical School, Essen, Germany; Department of Visceral Surgery, Marienhospital Herne, Ruhr University of Bochum, Medical School, Herne, Germany; Institute of Pathology, Ruhr-University of Bochum, Medical School, Bochum, Germany

**Keywords:** Human endogenous retroviruses, Chemotherapy resistance, Antiviral drugs

## Abstract

**Background:**

Endoretroviruses account for circa 8 % of all transposable elements found in the genome of humans and other animals. They represent a genetic footprint of ancestral germ-cell infections of exoviruses that is transmittable to the progeny by Mendelian segregation. Traces of human endogenous retroviruses are physiologically expressed in ovarial, testicular and placental tissues as well as in stem cells. In addition, a number of these fossil viral elements have also been related to carcinogenesis. However, a relation between endoretroviruses expression and chemoresistance has not been reported yet.

**Methods:**

Twenty colorectal carcinoma patient samples were scrutinized for HERV-W_E1_ and HERV-FRD_1_ endoretroviruses using immunohistochemical approaches. In order to search for differential expression of these elements in chemotherapy refractory cells, a resistant HCT8 colon carcinoma subline was developed by serial etoposide exposure. Endoretroviral elements were detected by immunocytochemical staining, qPCR and ELISA. IC_50_-values of antiviral and cytostatic drugs in HCT8 cells were determined by MTT proliferation assay. The antivirals-cytostatics interaction was evaluated by the isobologram method.

**Results:**

In this work, we show for the first time that HERV-W_E1_, HERV-FRD_1_, HERV-3_1_, and HERV-V_1_ are a) simultaneously expressed in treatment-naïve colon carcinoma cells and b) upregulated after cytostatic exposure, suggesting that these retroviral elements are intimately related to chemotherapy resistance. We found a number of antiviral drugs to have cytotoxic activity and the ability to force the downregulation of HERV proteins in vitro. We also demonstrate that the use of different antiviral compounds alone or in combination with anticancer agents results in a synergistic antiproliferative effect and downregulation of different endoretroviral elements in highly chemotherapy-resistant colorectal tumor cells.

**Conclusions:**

Enhanced HERV-expression is associated with chemoresistance in colon carcinomas which can be overcome by antiviral drugs alone or in combination with anticancer drugs. Therefore, the introduction of antiviral compounds to the current chemotherapy regimens potentially improves patient outcomes.

**Electronic supplementary material:**

The online version of this article (doi:10.1186/s13046-015-0199-5) contains supplementary material, which is available to authorized users.

## Background

Cancer chemotherapy is likely to be associated with the development of cancer stem cell-like phenotypes. This chemical stress can force the genesis of cell heterogeneity in the tumor that becomes manifest in its histology, protein expression pattern, and genetic/epigenetic signature [1]. In addition, it is known that drug or ionizing radiation exposure can induce the expression of viral elements present in the cells [2, 3]. However, the relationship between the manifest endoretroviral spectrum and the development of chemotherapy resistance has not been concatenated until now.

Accounting for 8 % of the human genetic material, human endogenous retroviruses (HERVs) represent a footprint of ancestral germ-cell infections in which viruses integrated into the host genome and were transmitted in a Mendelian form to the progeny [4]. Structurally, HERVs retain all retroviral hallmarks, including the *gal*, *pol & env* genes flanked by non-coding long terminal repeats (LTRs). Although most HERVs have lost the capacity of horizontal transmission due to gene defects, some have retained this ability despite their apparent apathogenicity [5–7]. To ensure proliferation, they sequestrate intact elements from co-expressed exoviruses to form functional entities [8–10].

While organs like ovaries and testes as well as embryonic stem cells express HERV elements abundantly, expression is typically low or non-detectable in somatic cells. Furthermore, it is known that HERV-W significantly contributes to the differentiation of cytotrophoblasts into syncytiotrophoblasts through the fusogenic properties of the syncytins (HERV-W_E1_ & HERV-FRD), which are products of the viral envelope gene [11–18].

So far, the contribution of HERVs to normal cell physiology remains largely unstudied. On the other hand, a number of fossil HERVs have been linked to neoplastic transformation that gives rise to breast and small-cell lung carcinomas, renal carcinomas, leukemias, and other malignancies [5, 19–21]. For example, the overexpression of HERV-H and HERV-V-3 was found to be correlated with the development of colon carcinoma, although any relationship to chemotherapy resistance or tumor aggressiveness has not been reported so far [22, 23].

It was recently demonstrated that iRNA targeting HERV-K can suppress tumor growth in melanoma models, suggesting that the overexpression of particular HERVs may play a crucial role in tumor physiology [24]. Consequently, interference with these viral elements via antiviral agents could produce antitumoral effects. The introduction of antiviral drugs such as ribavirin into the therapy of tumors with high HERV expression (e.g. refractory AML) has shown complete and partial responses and a reduction in overall levels of eIF4E [25–29]. Nevertheless, the influence of antiviral agents on the expression of these viral elements and their potential anticancer activity has not been reported yet.

Here, we show that cytostatic stress induces the development of highly resistant, HERV-overexpressing tumor cells. We determine the cytotoxic activity of different antiviral agents and highlight their capacity to shut down HERV expression. Finally, we demonstrate that the combination of antiviral compounds and antitumoral drugs reflects synergistic antiproliferative effects in highly resistant, HERV-overexpressing colorectal tumor cells.

## Materials and methods

### Cell cultures and patient samples

HCT8 colon carcinoma cells employed in this study were obtained from the cell and tumor bank of the University of Duisburg-Essen, Medical School. Mononuclear cells (MNC) were isolated from whole blood using Ficoll (Sigma-Aldrich, Missouri, USA) gradient following the manufacturer’s instructions. CD34+ cells were isolated using magnetic bead kits (Milteny, Cologne, Germany) following the kit instructions.

#### Patient samples

##### Ethical considerations

This study was reviewed and approved by the Committee on Ethics of the Ruhr-University of Bochum, Medical School (register numbers: 4042-11 and 5235-15). Written informed consent was obtained from each participant. Informed written consent regarding eligible subjects below 18 years was obtained from parents. Samples were anonymised, coded and accessible only by research staff. All patient samples were gathered by the division of visceral surgery, Marienhospital Herne, Germany. The histopathological of samples were performed by the institute of pathology of the Ruhr-University of Bochum, Medical School.

### IC_50_ values and induction of etoposide resistance

IC_50_ values were determined using the MTT [3-(4,5-dimethylthiazol-2-yl)-2,5-diphenyltetrazolium bromide] proliferation assay as described previously, and reported as the mean of three independent experiments. Briefly, cells in exponential growth phase were harvested, washed with medium, and seeded in 96-well plates at appropriate densities according to their growth kinetics. After a conditioning period of 24 hours, cells were exposed to increasing concentrations of cytostatics for 72 hours. The cultures were then incubated with MTT (Sigma-Aldrich, Munich, Germany) dissolved in PBS at a final concentration of 1 mg/ml for 4 hours. Supernatants were aspirated and the purple formazan crystals dissolved in 100 μl of solubilization solution (10 % SDS in DMSO, Sigma-Aldrich, Munich, Germany). The absorbance was measured in a microtiter plate reader (Infinite F200 Tecan, Berlin, Germany) at 570 nm. Both methods were formerly described [30, 31].

Resistance to etoposide in HCT8 cells was induced in the same form previously described [1, 30, 32]. Briefly, IC_50_ values for cytostatics were determined by MTT assay. Exponentially growing cells were then exposed to 2× IC_50_ for 24 hours. For recovery, cells were washed and incubated with drug-free culture medium until new colonies had formed. This procedure was repeated several times, each time doubling the original IC_50_ until 64× IC_50_ was reached. The surviving cells were subjected to a resistance selection by incubation with increasing concentrations of the respective drugs (16× to 512× IC_50_) for 24 hours. Cells which proliferated at higher drug concentrations (128×) within one week were considered chemotherapy refractory. Resistant colonies were then expanded in the continuous presence of cytostatics and used for molecular-biological analysis, in particular for studying the expression of CSC features. The resistance factor (RF) was determined by MTT proliferation assay and reported as the IC_50_ iCSCs/ IC_50_ parental ratio. Using etoposide as chemoresistance-inducer it is feasible to induce a wide HCT8 subpopulation of cells (HCT8^RETO^) with cancer stem cell features (CSCs) in a very short time. HCT8^WT/RETO^ cells were cultured in DMEM medium (Biochrom, Berlin, Germany) containing 10 % heat-inactivated fetal calf serum (FCS) and 15 μg/ml Ciprobay (Bayer AG, Wuppertal, Germany).

### Studies on the expression of human endogenous retrovirus elements (HERVs) in colorectal carcinomas (CRCs)

We analyzed the expression of HERV-_WE1_ and HERV-_FRD1_ in patient samples as well in HCT8^WT/RETO^ colon carcinoma cell line using both immunocytochemical (ICC) and immunohistochemical (IHC) staining.

ICC and IHC staining was performed according to standard protocols [1]. Briefly, ICC cells were grown in chamber slides to appropriate densities, washed with 1× PBS, fixed with 4 % formaldehyde in PBS for 20 minutes, rinsed twice with 1× PBS for 5 minutes, and blocked with 10 % normal goat serum (AbD Serotec, London, UK) at room temperature for 60 minutes. For IHC, tissue samples were fixed with 4 % formaldehyde in PBS and embedded in paraffin. Paraffin tissue sections of 4 μm thickness were baked overnight at 60 °C to firmly attach the sections to the slides. After baking, the sections were deparaffinized in 2 changes of xylene-substitute (Thermo Scientific, London, UK) solution for 10-15 min and rehydrated in a series of graded ethanol solutions (100 %, 100 %, 95 %, 70 %, 50 %) for 3 minutes each. HE staining was performed using conventional techniques. For IHC, antigens were retrieved by heating the sections for 30 minutes in 10 mM sodium citrate buffer pH 9.0 at 95 °C in a domestic vegetable steamer. The slides were washed twice in 1 × PBS for 5 minutes and blocked for 60 minutes with 10 % normal goat serum at room temperature. Primary antibodies (Bioss Antibodies, Woburn, USA and Biorbyt, Cambridge, England) were applied overnight according to the manufacturers' recommendations. On the next day, the slides were washed 3 times in PBST (PBS/0.05 % Tween 20) for 5 minutes each and rinsed in 1 × PBS for another 5 minutes. Conjugated secondary antibodies (Cell signaling, Cambridge, UK) diluted in PBS/0.05 % Tween 20/2.5 % goat serum were incubated for 120 minutes at room temperature according to the manufacturers' recommendations. Next, the samples were stained for 15 minutes with 1 μg/ml Hoechst 33258 diluted in PBS in order to visualize the nuclei. The slides were then washed 3 times in PBST (PBS/0.05 % Tween 20) for 5 minutes each and rinsed in 1 × PBS for another 5 minutes. Tissue specimens were mounted in Faramound Mounting medium (Dako) for visualization.

### Differential expression of HERV transcripts in HCT8^WT/RETO^ colon carcinoma cells

#### RNA purification and cDNA synthesis

Total RNA was extracted with Trizol® (Life Technologies, California, USA). To eliminate genomic DNA contamination, the eluted RNA containing 10 IU RNase inhibitor was treated with 7 Kunitz units of RNase-free DNase I (Qiagen, Hilden, Germany) in the appropriate buffer and incubated at 25 °C for 20 minutes. The RNA samples were then purified further on RNeasy mini columns (Qiagen, Hilden, Germany) according to the manufacturer’s instructions. RNA integrity was ascertained by agarose gel electrophoresis and densitometric analysis. 1 μg of pure and intact RNA was used for first-strand cDNA synthesis using the cDNA Reverse Transcription Kit from Life Technologies, following the kit instructions.

### qPCR

HERV expression was monitored by qPCR with validated primers and probes from Life Technologies (Cat. Nr.: 18S Hs99999901_s1, HERV WE1 Hs01926764_u1, HERV-FRD1 Hs01942443_s1, HERV3-1 Hs 04184598_s1 and HERV-V1 Hs00708335_s1), using the Taqman PCR core reagents according to the manufacturer’s recommendations. In addition, the expression of these HERV-elements was confirmed using specific primers purchased from Biomol (Hamburg, Germany). The primers details are reflected in Table 1. The amplification of 25 ng of RNA was performed in triplicate in a CFX96TM Real-Time System (Biorad Laboratories, California, USA). Results were analyzed with CFX-ManagerTM Software Version 3.1 (Biorad Laboratories, California, USA). The evaluation of HERV relative expression was determined using the Ct comparative method.

**Table 1:** Real Time PCR primers used for the detection of HERVs. The accession, region, sequence, polarity and product size for the primers used are reflected

Symbol	Accession	Region	Forward	Reverse	Size _(bp)_
18S	NR003286	1025-1513	tcaagaacgaaagtcggagg	ggacatctaagggcatcaca	488
HERV-W_E1_	AF072506	290-463	gggttccatggttctcttct	tggtgaaccacttccaagat	174
HERV-FRD_1_	NM207582	504-698	ctcattctcacgccttcact	taattccgcctctatgcttg	195
HERV-V_1_	NM152473	1565-1757	gggcaaagattctgcaacta	ttgtctggctacctgcctac	193
HERV-3_1_	NM001007253	1377-1562	taaccagaaattgcctgagc	gaagaggcggttagtgtgaa	186

### Analysis of the simultanean interaction of antiviral and cytostatic drugs

Amantadine, ribavirin, pleconaril, lamivudine, and doxorubicin were purchased from Sigma-Aldrich, acyclovir and ganciclovir from HEXAL AG, Holzkirchen, Germany. Retrovir was obtained from ViiV Healthcare, London, UK, Foscavir from Clinigen Healthcare, Staffordshire, UK and brivudine from Berlin Chemie, Germany. Etoposide and cisplatin were purchased from TEVA GmbH and 5FU from Medac, both Hamburg, Germany.

The simultaneous effect of antiviral drugs and cytostatics was analyzed by the isobologram method (50 % isodose) as described previously [30]. Briefly, the IC_50_ for both substances were first determined using the MTT proliferation assay. Applying fixed percentages of the IC_50_ for the first drug (20, 40, 60, 80 and 100 %) and varying the concentration of the second drug from 0.1 to 50 μM, the variation in the resulting IC_50_ was determined for every percentage. The same procedure was carried out inversely for the second drug. Dose-response curves were then plotted and evaluated.

#### Protein isolation and Western blot analysis

To evaluate the direct effect of antiviral drugs on the expression of HERV proteins we exposure HCT8 cells to amantadine, pleconaril and ribavirin alone or simultaneously at 1-fold their respective IC_50_-values for 24 hours.

3 × 10^6^ HCT8^WT/RETO^ cells growing exponentially in 75 cm^2^ TC flasks were incubated in medium containing the respective IC_50_ of amantadine, pleconaril, and ribavirin alone or with all drugs simultaneously for 24 hours. Medium was then removed and the cells washed twice with cold PBS. Protein extraction was performed using RIPA buffer as previously described [1]. Briefly, pellets were lysed in RIPA buffer [150 mM NaCl, 1 mM EDTA, 1 % Triton X-100, 1 % sodium deoxycholate, 0.1 % SDS, 50 mM Tris-HCl pH 7.4] in the presence of a proteinase inhibitor cocktail according to the manufacturer’s instructions (Roche Diagnostics GmbH, Mannheim, Germany) for 30 minutes on ice and then centrifuged for 20 minutes at 14 000 g, 4 °C. The homogenates were measured for protein content using Bradford and normalized to the same protein concentration. Protein extracts (30 μg) were resolved by SDS-PAGE in a 4–12 % gradient gel (Invitrogen, Karlsruhe, Germany) using Tris-glycine (0.025 M Tris-HCl, 0.192 M glycine pH 8.5) buffer, and transferred overnight to 0.2 μm nitrocellulose membrane (Pierce Protein, Thermo Scientific Inc., MA, USA). Blots were blocked with 5 % BSA or non-fat milk taking into consideration the recommendations of the manufacturers of the primary and secondary antibodies. Primary antibodies were purchased from Bioss Antibodies, Woburn, USA. Conjugated secondary antibodies were obtained from Cell Signaling and Jackson ImmunoResearch Europe Ltd. (Suffolk, UK). Immunoblots were developed by Western Lightning® Plus-ECL (Perkin Elmer, CA, USA) using a ChemiDoc XRS+ system with Image Lab Version 2.0.1 software (Biorad, CA, USA).

### Enzyme-linked immunosorbent assay (ELISA)

Differential HERV expression and its repression by antiviral drugs were monitored using an indirect ELISA method. In brief, 96-well microtiter plates (Greiner Bio-One GmbH, Frickenhausen, Germany) were coated with protein homogenates (5 μg/100 μl) overnight at 4 °C. Well contents were aspirated and the wells washed 3 times with washing buffer (PBS/0.05 % Tween 20). The wells were then incubated with 300 μl blocking buffer [PBS/0.05 % Tween 20/1 % bovine serum albumin (BSA)] each at 37 °C for 1 h and then washed 3 times. Primary antibodies diluted 100 μl in blocking buffer 1:500 were added, followed by incubation at 37 °C for 1 h. The wells were aspirated and washed three times followed by incubation with an HRP-conjugated secondary antibody (Sigma-Aldrich) in 100 μl at 37 °C for 1 h, dilution 1:2000. The wells were washed 3 times and incubated at 37 °C for 30 min with 100 μl of fresh 0.4 mg/ml *o*-phenylenediamine and 0.4 mg/ml urea/H_2_O_2_ dissolved in 0.05 M Na_2_HPO_4_/0.05 M citric acid adjusted to pH 5. The color reaction was stopped with 50 μl of 1 M HCl per well, and the optical density measured after 1 h at 492 nm (OD_492_) on an Infinite M200 microtiter plate reader (Tecan, Maennedorf, Switzerland). Results were normalized using beta-actin as control and presented as percent of expression.

### Statistical analysis

Experiments were performed at least in triplicate and the data given as means ± standard error of means (SEM), unless stated otherwise. Student’s t-test with four degrees of freedom was used to compare independent groups. The statistical analyses were performed with Sigma Plot 12 (Systat Software Inc., California, USA). A probability (p) value was considered *: significant (*p* < 0.05); **: very significant (*p* < 0.01); ***: highly significant (*p* < 0.001). ICC and IHC microscopy studies were descriptive and therefore not analyzed statistically; the results shown are representative of at least *n* = 3 independent experiments.

## Results

### HERV expression in colorectal carcinoma patient material

**Fig. 1:**
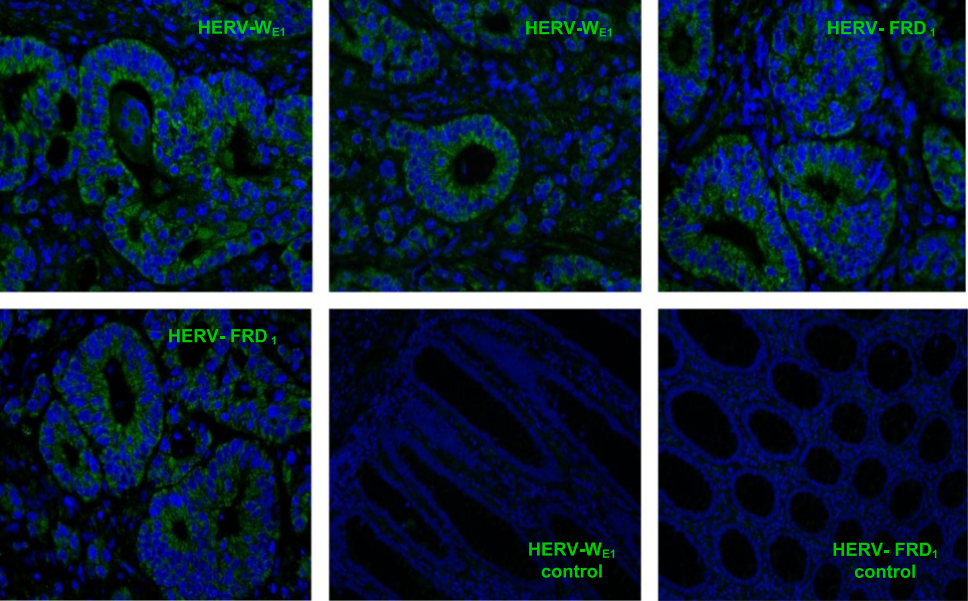
Overexpression of HERV-W_E1_ and HERV-FRD_1_ in colon adenocarcinoma patient sections analyzed by IHC. Both elements are principally expressed in the villi, intervillar space and crypts of the large intestine especially in the tumor area, normal tissues are almost negative. Magnification 400x, *n* = 20 independent sections

The expression of the HERV elements W_E1_ and FRD was monitored by IHC in human colorectal adenocarcinoma paraffin sections. Both elements were found to be overexpressed in tumors as compared to the normal colon tissues. Fig. 1 shows that the expression of both viral traces was localized to the villi, intervillar regions and the crypts of the large intestine, but confined to the tumor areas.

### Overexpression of HERV-W_E1_ and HERV-FRD_1_ in HCT8^RETO^ colorectal carcinoma cells

The anticancer drug etoposide is commonly not employed in the treatment of colorectal carcinomas (CRC). Nevertheless, this drug is associated with the induction of cancer stem cell characteristics in several tumors as previously described. In HCT8 colorectal carcinoma cells, etoposide induces characteristics for stemness, as judged by their self renewal capacity, colonosphere formation, radioresistance and markedly reduced chemotherapy sensitivity as well as a defined set of CSC-markers like EpCAM, Mucins 1 & 4, c-Myc, Stat3, stem cell factor (SCF), Musashi, β-catenin, among others (*see* Additional file 1). These cells showing CSC-features were designated as HCT8^RETO^ cells.

**Fig. 2:**
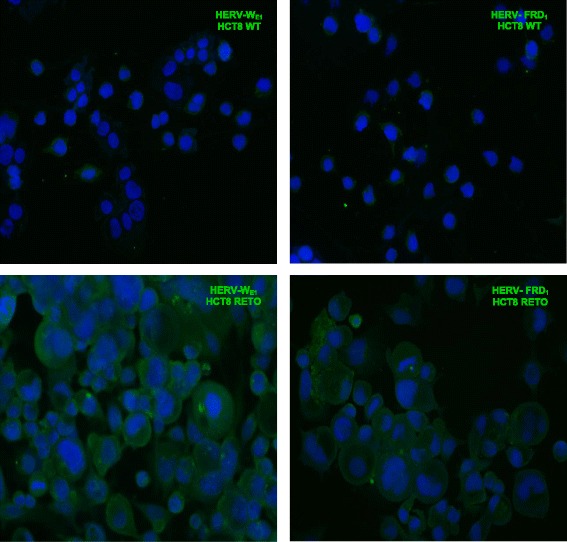
Differential expression of HERV-W_E1_ and HERV-FRD_1_ in HCT8^WT/RETO^ colon carcinoma cells analyzed by ICC. Cells growing on chamber slides were labeled with anti-HERV-W_E1_ and anti-HERV-FRD antibodies. Wildtype cells express basal levels of both HERV elements while the HCT8^RETO^ cells express high levels and develop hypertrophy. Magnification 400x, *n* = 3 independent experiments

We investigated the differential expression of HERV-W_E1_ and HERV-FRD_1_ in colon adenocarcinoma based on the cell line HCT8^WT/RETO^. During development of resistance against etoposide, the first observable change was the emergence of a transitory cell hypertrophy as depicted in Fig. 2, together with the overexpression of several endoretroviral elements as observed by ICC.

### Various HERVs transcripts are simultaneously expressed in HCT8^WT^ and overexpressed in HCT8^RETO^ cells

**Fig. 3:**
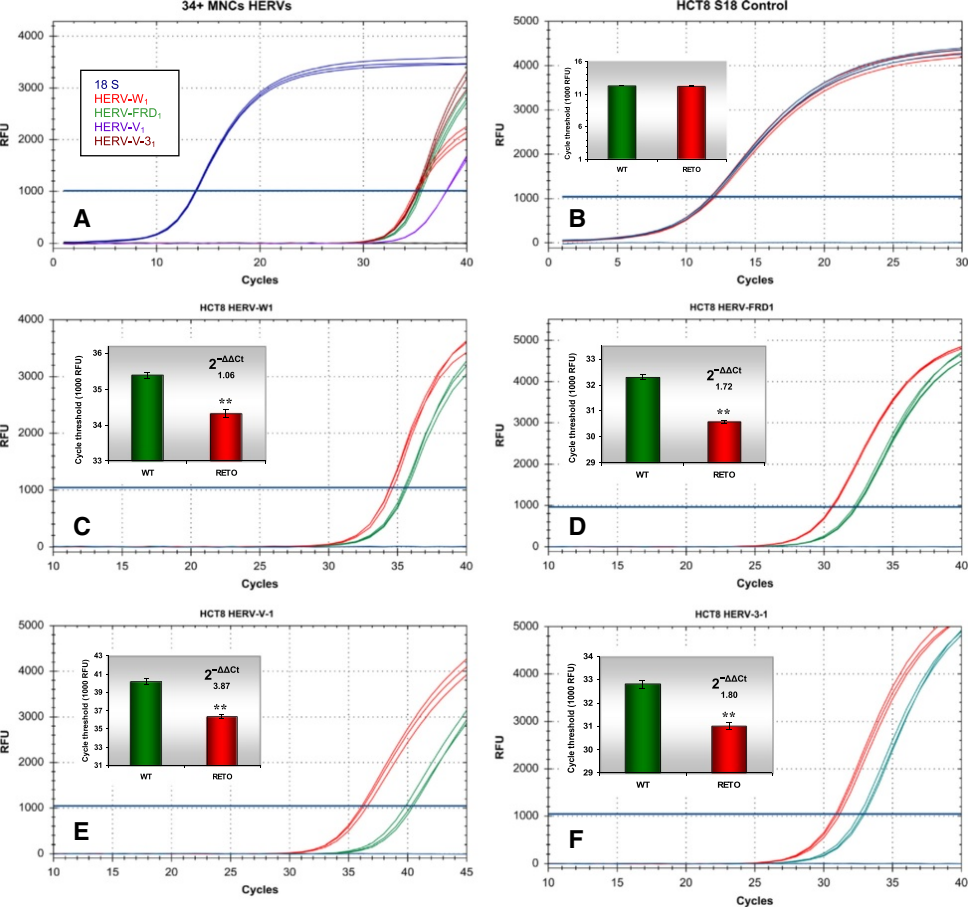
Differential expression of HERVs in HCT8^WT/RETO^, analyzed by real-time PCR. **a**: Amplification pattern of different HERVs in CD34^+^ mononuclear cells (MNC) from umbilical blood. The cells express all analyzed HERV variants. **b**: 18S endogenous control. **c**: Differential expression of HERV-WE_1_ (2^−ΔΔCt^ 1.06). **d**: Degree of difference of HERV-FRD_1_ (2^−ΔΔCt^ 1.72). Degree of difference of HERV-V_1_ (**e**, 2^−ΔΔCt^ 3.87) and HERV 3_1_ (**f**, 2^−ΔΔCt^ 1.80) transcripts in HCT8 ^WT/RETO^. In HCT8^RETO^, all HERV are overexpressed compared to HCT8^WT^. Red, WT; green, RETO, *n* = 3 independent experiments

Human stem cells have been described to express a range of different HERVs. It is also known that these elements, especially HERV-H, are expressed in colon carcinomas, probably due to the presence of cancer stem cells. The expression of different HERVs and a possible relationship to chemosensitivity has not been reported yet. For this purpose, we analyzed HCT8^WT/RETO^ cells for HERV mRNAs. Mononuclear CD34^+^ cells from umbilical cord blood served as positive control since these cells were found to co-express a battery of HERV elements. It was striking, that expression of HERV mRNA transcripts WE_1_ (Fig. 3c), FRD_1_ (Fig. 3d) 3_1_ (Fig. 3e) and V_1_ (Fig. 3f) was significantly enhanced in HCT8^RETO^ cells, compared to parental HCT8^WT^ cells (range from 1.06 to 3.87-fold).

We next asked whether incubation with antiviral compounds might have an impact on tumor biology and/or proliferation of chemorefractory cancer cells *in vitro*.

### Enhanced cytotoxic effect of antiviral compounds on chemoresistant tumor cells in vitro

**Fig. 4:**
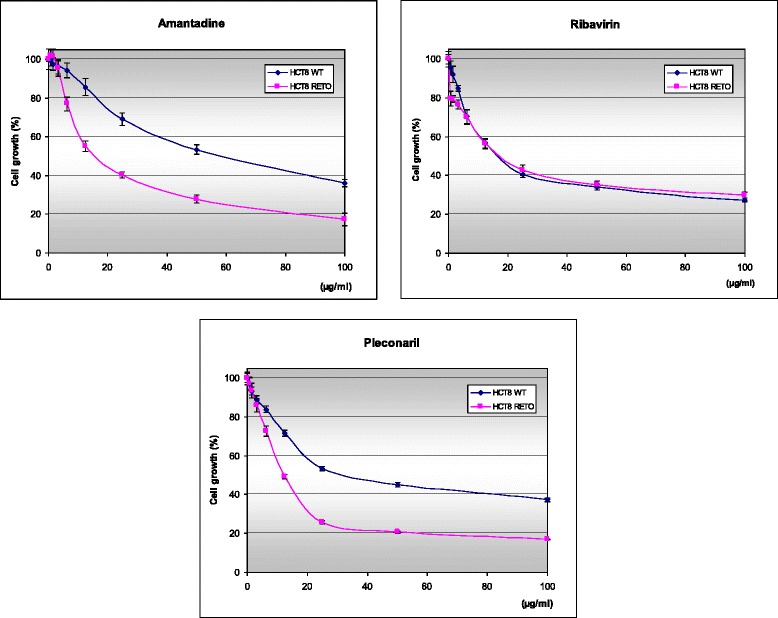
*In vitro* cytotoxicity of amantadine, ribavirin and pleconaril in HCT8^WT/RETO^ cells. Cytotoxicity was measured over 24 hours by MTT assay. Amantadine and pleconaril show a specific activity in the highly chemotherapy-resistant subline. Values represent the means ± standard deviation of at least *n* = 3 independent experiments

To get a first hint on the antiproliferative activity of antiviral compounds in colon carcinoma cells, we analyzed antiviral drugs like amantadine, ribavirin, pleconaril, lamivudine, acyclovir, ganciclovir, AZT, foscavir and brivudine in HCT8^WT/RETO^ cells. Of these, only three compounds showed some degree of antiproliferative effect, as defined by their IC_50_ values: amantadine, ribavirin and pleconaril (Fig. 4). All other drugs did not show detectable activity at concentrations up to 200 μg/ml (data not shown) and therefore were excluded from further analysis. In general, amantadine and pleconaril were the most active compounds in the HCT8^RETO^ cells (Fig. 4).

### Synergistic cytotoxic effects between amantadine and anticancer drugs in HCT8^WT/RETO^ cells

We next questioned the potential interaction between antiviral compounds and classical, clinically used anticancer drugs *in vitro*. In this work results were only shown for amantadine, but similar data were also achieved for pleconaril and ribavirin, respectively.

**Fig. 5:**
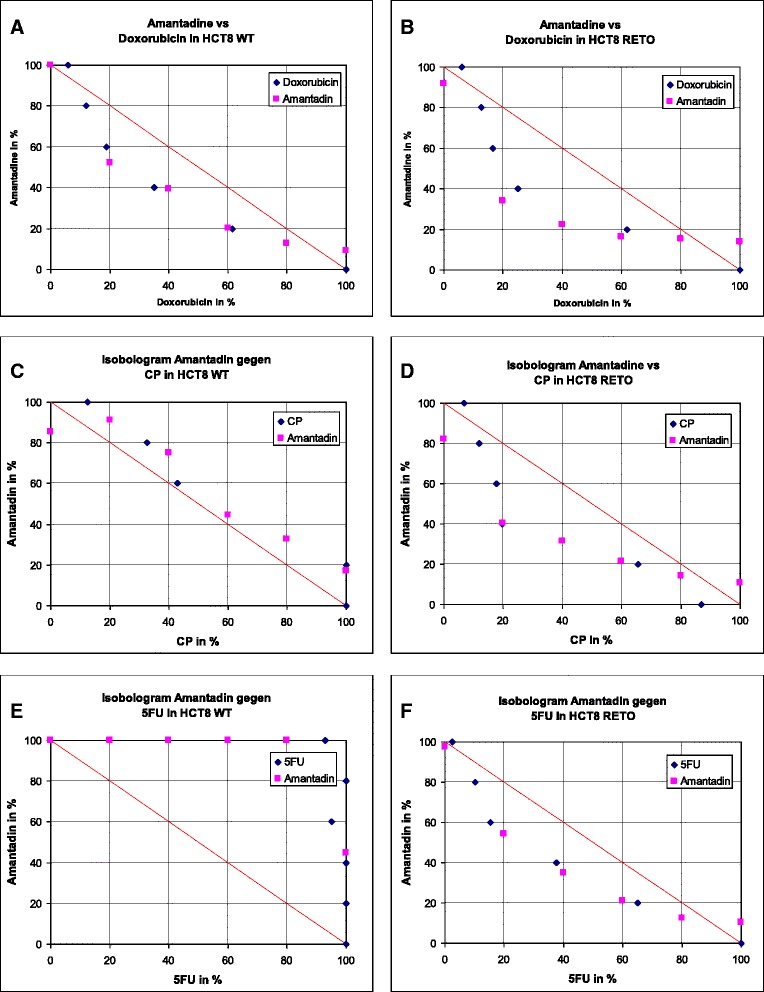
Isobologram showing the *in vitro* interaction between amantadine and doxorubicin, cisplatin or 5FU in HCT8^WT^ and HCT8^RETO^ cells. Dose-response curves are based on the respective IC_50_ values calculated after 24 hours of incubation. Amantadine-doxorubicin are synergistic in HCT8 ^WT/RETO^ (**a & b**). Amantadine-cisplatin are additive in HCT8 wildtype (**c**) and synergistic in HCT8^RETO^ (**d**). Amantadine-5FU are synergistic in RETO (**f**) but antagonistic in HCT8^WT^ (**e**). *n* = 3 independent experiments

In general, using the chemo-sensitive HCT8^WT^ cell line (Fig. 5, panels a, c and e), interaction between amantadine and doxorubicin was slightly synergistic (panel **a**), between amantadine and cisplatin (CP) additive (panel **c**), and remarkable antagonistic activity was observed between amantadine and 5FU (panel **e**), respectively. In contrast, in the chemoresistant HCT8^RETO^ cell line (Fig. 5, panels b, d and f), synergistic interaction between amantadine and all three anticancer drugs was shown, although synergism was less pronounced using 5-fluorouracil (5FU) (panel **f**). These results indicate that antiviral compounds like amantadine do not only exhibited substantial cytotoxic activity in chemoresistant tumor cells by itself, but also enhance the cytotoxic efficacy of anticancer drugs *in vitro*.

**Fig. 6:**
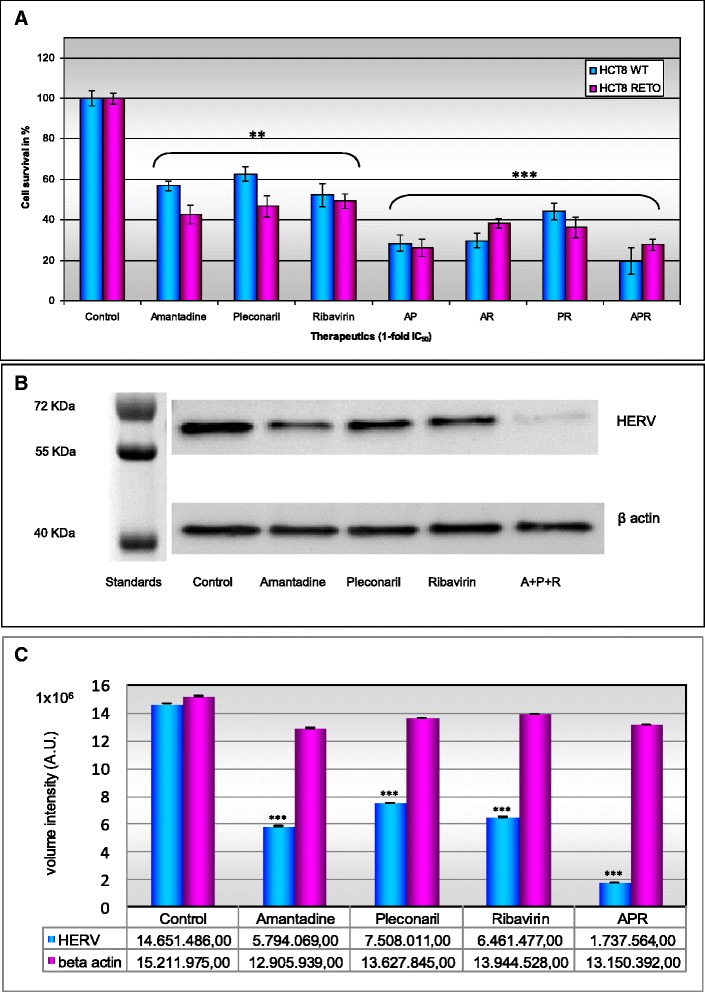
Cytotoxic effect of antiviral drugs and their influence in the expression of HERV proteins in HCT8 colon carcinoma cells. Simultaneous incubation of HCT8^WT/RETO^ with amantadine, ribavirin and pleconaril based on the IC_50_ values calculated for the drugs applied alone or in combination for 24 hours. **a**: Influence of Amantadine, Pleconaril and Ribavirin in the expression of HERVs analyzed by ELISA in HCT8^WT/RETO^ cells. **b**: Down regulation of HERVs proteins after antiviral exposure analyzed by Western blot. **c**: relative expression of HERVs under the influence of antiviral drugs. Amantadine-pleconaril show a potentiation of circa 35 % in HCT8^WT^ and 20 % in HCT8^RETO^. Amantadine-ribavirin have a potentiation of circa 25 % in HCT8^WT^ and 10 % in HCT8^RETO^. The three-way combination ARP was the most effective. Results are representative of *n* = 3 independent experiments

We also explored the potential synergistic antiproliferative effects by combining different antiviral compounds by using their specific IC_50_. As expected, amantadine, pleconaril and ribavirin as single drugs reduced cell survival by about 50 %. Enhanced cytotoxicity in HCT8^RETO^ cells was only detectable for amantadine and pleconaril. In general, combination of antiviral compounds (Fig. 6, panel b) resulted in enhanced cytotoxicity, especially for amantadine-containing doublets or even triplets. However, all antiviral combinations did not show enhanced activity in chemo-resistant HCT8^RETO^ cells, compared to chemo-sensitive HCT8^WT^ cells, indicating unspecific cytotoxic effects of antiviral compounds rather than additional suppression of HERV-protein expression.

In addition we analyzed if the antiviral compounds alone or applied as polychemotherapy exerts effects on the regulation of HERVs proteins in HCT8^RETO^ cells, which abundantly expressed different HERVs entities. We found that amantadine, pleconaril and ribavirin alone or in combination applied simultaneously, significantly downregulated the protein expression of HERVs as reflected in Fig. 6, panel b. This effect was drastically observed for the simultanean incubation of all antivirals studied (Fig. 6, panels b & c). These results certainly demonstrated that cytotoxicity of antiviral compounds in chemoresistant HCT8RETO cells is also associated with downregulation of HERV-proteins.

### Downregulation of HERV-proteins upon exposure of chemo-resistant HCT8^RETO^ cells to the antiviral compound amantadine

**Fig. 7:**
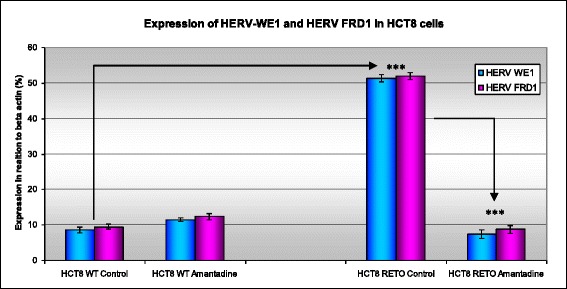
Effect of amantadine on HERV protein expression in HCT8^WT/RETO^ analyzed by ELISA. The expression of both HERV-W_E1_ and HERV-FRD_1_ is downregulated after incubation with amantadine at 2 x IC_50_ for 24 hours. Expression is reduced to basal levels in HCT8^RETO^. The results are given in percent in relation to the expression of beta-actin as endogenous control

We have already shown enhanced cytotoxicity of antiviral compounds in chemoresistant HCT8^RETO^ cells in vitro (Fig. 4). Furthermore, we shown that amantadine is able to reduce significantly the HERV protein expression at 1-fold its IC_50_ as observed by Western blotting (Fig. 6, panel b & c). Fig. 7 depicts the semi-quantification of the effect of amantadine in HCT8^WT/RETO^ cells as assayed by ELISA. As expected, the protein expression of HERV-W_E1_ and FRD_1_ is markedly enhanced in HCT8^RETO^ cells (panel 3) compared to HCT8^WT^ cells (panel 1). Upon incubation with amantadine, pronounced reduction of HERV-W_E1_ and -FRD_1_ protein expression was only detectable in HCT8^RETO^ cells. Comparable data were achieved for HERV-W_E1_ and FRD_1_ at the RNA levels (data not shown).

## Discussion

In this study, we have demonstrated that enhanced expression of various HERV proteins is not only detectable in colon cancer cells, but might also have therapeutic implications for CRC patients especially in chemorefractory tumors.

A number of HERVs have been found to be upregulated during carcinogenesis in tumors derived from tissues that normally show no or only basal expression of these elements. For example, HERV-K transcripts of the *Env* protein, while entirely absent in normal breast tissue, were demonstrated to be overexpressed in almost all breast carcinomas [33–35]. In addition, the expression of HERV-H and HERV-3-1 in colon carcinomas has been reported [22, 23].

Recently, we described a method to induce multi-resistant cancer cells that express several CSC tissue-related markers as well as stemness features like sphere formation, radio- and chemoresistance [1, 32]. These cells also showed an up-regulation of a set of HERVs. Moreover, HERVs expression has been also linked to stemness in both normal and cancer cells [36].

Colon adenocarcinoma paraffin sections showed significant expression of HERV-W_E1_ and HERV-FRD_1_. These viral transcripts, as well as HERV 3_1_ and HERV V_1_, were also expressed in HCT8^WT^ and overexpressed up to three times in chemotherapy refractory HCT8^RETO^ cells, suggesting a relationship to chemoresistance. Consequently, additional HERV elements might contribute to carcinogenesis and chemotherapy resistance [4, 5, 10, 11, 21].

So far, a possible relationship of HERVs with chemotherapy resistance might be a result of the interaction of these proteins with cell membrane structure. Hypothetically, the ability of HERV-W_E1_ and HERV-FRD proteins to promote cell-cell fusion and generation of multinucleated giant cancer cells could represent an alternative membrane-mediated defense mechanism [12, 14, 15, 37, 38]. Moreover, HERV overexpression could serve as a benchmark to monitor therapy resistance.

The first evidence for the impact of HERV gene repression on the inhibition of tumor cell growth came from the group of Thierry Heidmann [24], who used iRNA directed against HERV-K in a melanoma model. Wang-Johanning and coworker reported on the immunotherapeutic potential of anti-human endogenous retrovirus-K envelope protein antibodies in targeting breast tumors *in vitro* and *in vivo*. These anti-HERV-K-specific monoclonal antibodies inhibited tumor growth and induced apoptosis of breast cancer cells [39].

These data served as a rationale to examine antiviral drugs for antiproliferative activity and downregulation of HERV proteins in a panel of HERV-expressing chemoresistant cancer cell lines. Among these compounds, the structurally unrelated amantadine, ribavirin and pleconaril were found to be the most active, with IC_50_ values below 20 μg/ml, that might be clinically relevant.

Amantadine is approved for use as antiviral and antiparkinsonian drug. No primary mechanism of action has been described so far, but it its known that its interference with Influenza virus protein M2 plays an important role in repressing both the early and late phase of viral replication cycle. We focused our studies on amantadine because we previously have been investigating several PPAP compounds (polycyclic polyprenylated acylphloroglucinols) such as nemorosone and plukenetione A, which structurally share the adamantane backbone. We already reported on the antitumoral and antiretroviral activity of these drugs, which we found to inhibit HIV. Moreover, PPAPs were recently described as selective agents in highly resistant neuroblastoma entities [40–43], exerting a pleiotropic effect that involves the downregulation of transcription factors which may interact with viral promoters like Myc, Myb and Stat1/3 [44].

The combination of amantadine with doxorubicin, cisplatin and 5FU acted synergistically in etoposide-refractory HCT8^RETO^, i.e. amantadine boosts the cytotoxicity of these cytostatics in resistant cells.

We also addressed the combination of amantadine, ribavirin and pleconaril on overall cytotoxicity in HCT8^WT/RETO^ cells. Enhanced efficacy was observed for the combinations AP (amantadine-pleconaril), AR (amantadine-ribavirin) and ARP (amantadine-ribavirin-pleconaril), the latter being the most cytotoxic.

In conclusion, our data suggest that enhanced expression of various HERV proteins might have therapeutic implications in colorectal cancer. Therefore, the introduction of antiviral compounds to the current chemotherapy regimens potentially improves patient outcomes.

## Additional file

Additional file 1:
**HERV-3 protein downregulation.** (DOCX 157 kb)
