# Supplementary material for: Therapeutic potential of antiviral drugs targeting chemorefractory colorectal adenocarcinoma cells overexpressing endogenous retroviral elements
Source: J Exp Clin Cancer Res. 2015 Aug 12;34(1):81. doi: 10.1186/s13046-015-0199-5 (PMC4542094; doi:10.1186/s13046-015-0199-5)
Supplement: Additional file 1: — HERV-3 protein downregulation. (DOCX 157 kb) [file 13046_2015_199_MOESM1_ESM.pdf]

Clonogenic culture and self-renewal capacity

Clonogenic culture and self renewal capacity was a modification of the agar system developed by Stephen P. Thomson and described extensively elsewhere. Single HCT8 iCS cells were seeded in 0.7% agar supplemented with DMEM and primary colonies were removed after 21 days. Primary spheres (**A**) were mechanically disaggregated into single cells and replated into agar until secondary spheres were formed (**B**). The cell viability was monitored incubating agar cultures with MTT at final concentration of 0.1 mg/ml (**C**). These *in vitro* procedures allowed a simple and direct assessment of the self-renewal capacity of spheres-forming colon carcinoma cells.

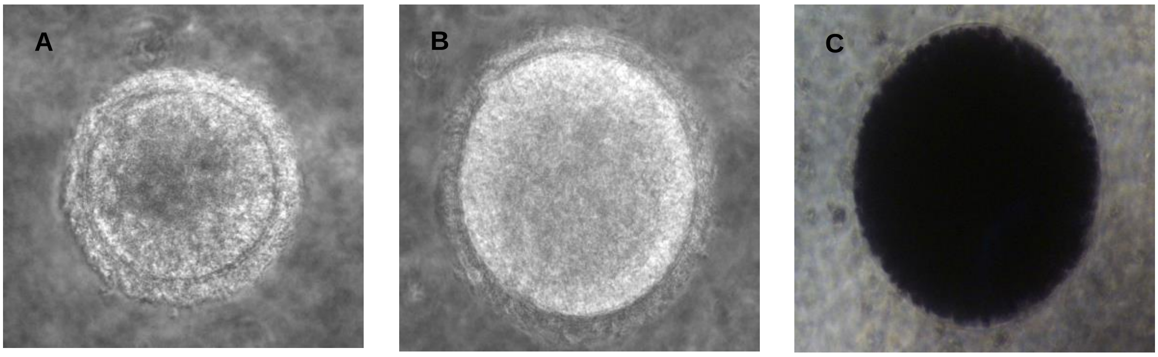

Figure 1: Colonospheres derived from HCT8 cells obtained in agar in serum-free DMEM. **A**, primary spheres after 21 days. **B**, secondary spheres derived from the mechanical disruption of primary cells. **C**, colonosphere viability assayed by MTT. Magnification 200x.

Ionizing irradiation of cell cultures and radioresistance studies

It is widely accepted that CSCs are both chemo and radioresistant. We addressed this issue in HCT8 iCSCs and noted that the induction of chemorefraction via etoposide derives in a concomitant radiorefraction as observed in **Figure 2**. Radioresistance was analyzed as previously reported (Díaz-Carballo et al.). In brief, cells were seeded in 96-well microtiter plates at a density of 250 to 1000 cells per well. After 24 h, cultures were irradiated at room temperature using a Pantak x-ray machine (Pantak) operated at 310 kV, 10 mA with a 2 mm AI filter (effective photon energy *90 kV), at a distance of 75 cm and a dose rate of 2.7 Gy/min ultrasoft x-rays for approximately a final dose of 20 Gy. Dosimetry was performed with a Victoreen dosimeter that was used to calibrate an in-field ionizing monitor. The culture plates were returned to the incubator immediately after irradiation; after 96 h, cell proliferation was determined by MTT assay.

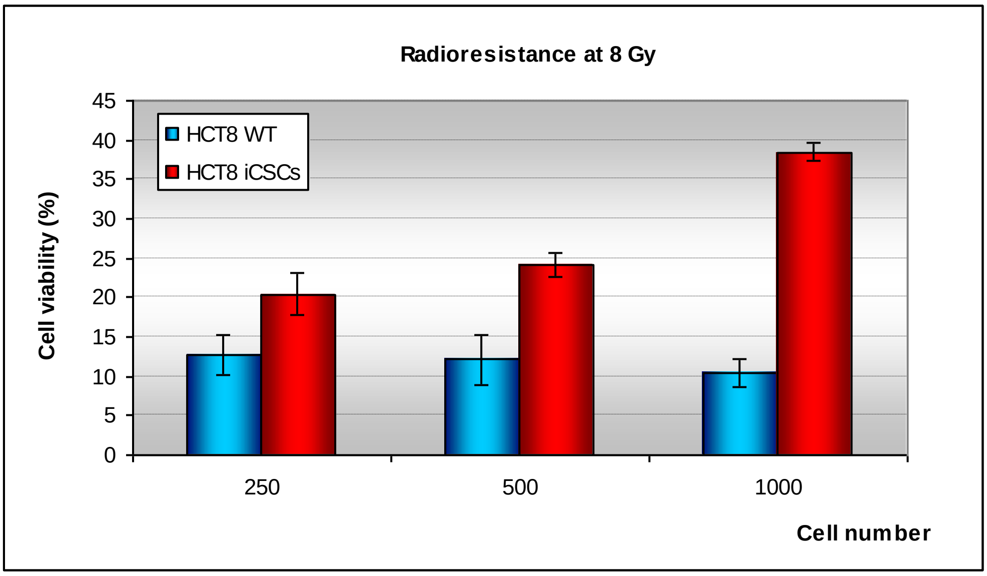

Figure 2: Radioresistance of a subset of HCT8 cells selected by etoposide exposure. Chemotherapy refractory subpopulations revealed a strong refraction to ionizing irradiation (8 Gy). Experiments are performed at least n = 3.

Induction of resistance via etoposide induces crossresistance with clinical relevant cytostatics

To induce chemotherapy resistance in vitro, several protocols mimicking clinical chemotherapy regimens have been designed. Induction of CSCs from tumor cell lines was performed by the selection of subpopulations using etoposide as previously described (Díaz-Carballo et al., 2014). We noted that resistant tumor cells selected via exposure to cytostatics evolved CSC features and crossresistance to several structurally unrelated cytostatics which remained stable even after xenografting. **Figure 3** depicts the crossresistance studies on iCSCs derived from HCT8 colon carcinoma cell line.

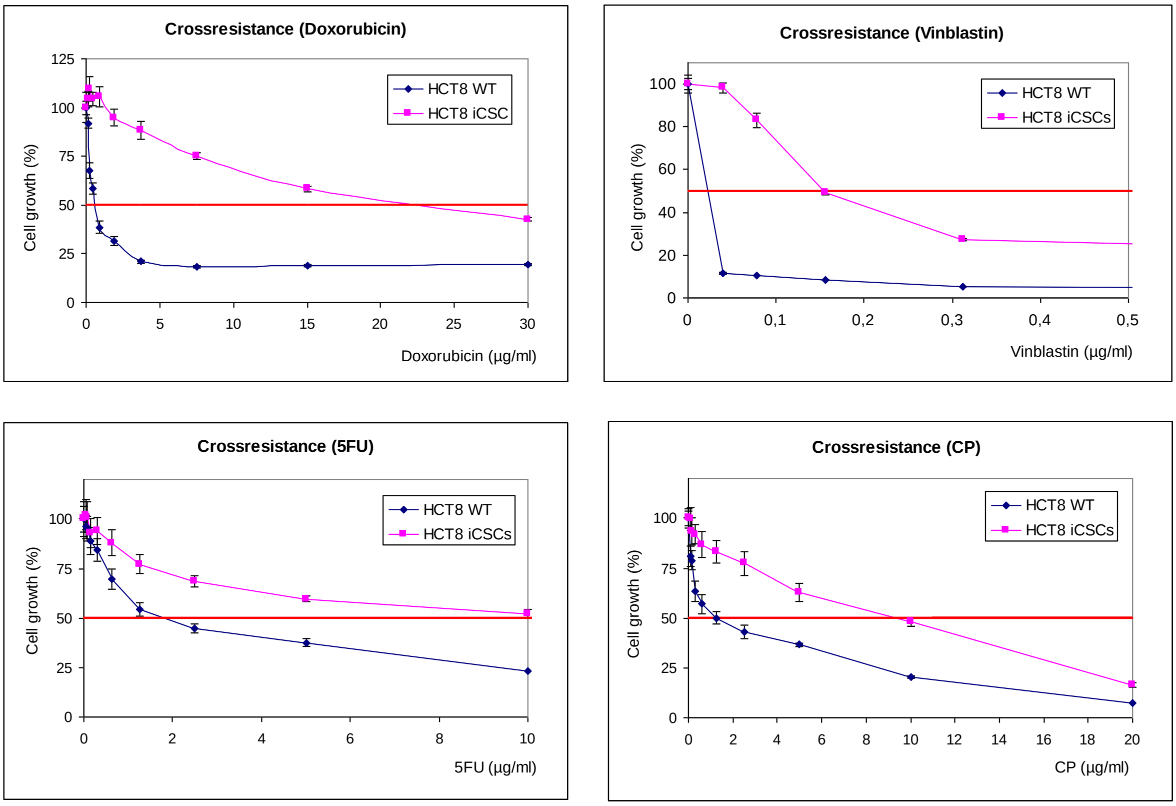

Figure 3: Crossresistance of a subset of HCT8 cells selected by etoposide exposure. Experiments are performed at least n = 3.

Induction of resistance via etoposide triggers expression of CSC phenotypes

In prior works we reported that resistant tumor cells selected via exposure to etoposide exhibit CSC-like features (iCSCs) which remained stable even after xenografting. Etoposide induces an entopic expression of several transcription factors involved in the gain of aggressive tumor phenotypes. Highly aggressive colon carcinoma tumors are characterized by the expression of markers associated with poor prognosis and CSC phenotype like as c-Myc, Stat 3, Ep-CAM, Mucins 1 & 4, CD44, CD133 and several signaling pathways. All these proteins were found upregulated in iCSCs derived from human colon adenocarcinoma HCT8 cells, proving a valid tool for phenotypic characterization of CSCs.

Transcription factors and cell proliferation effectors

c-Myc is a strong proto-oncogen constitutively expressed in several tumors and likewise a druggable driver gene, which is believed to govern the expression of a widespread of proteins implicated in chemotherapy resistance, cell cycle, signaling, apoptosis, cellular transformation and differentiation, as well as **pluripotency** among others (Zuqin Nie et al., Denis N et al.) c-Myc is aberrantly expressed in HCT8 iCSCs as shown in **Figure 4**.

Stat 3 (**Signal transducer and activator of transcription 3)** can promote oncogenesis by being constitutively active through various pathways i.e. JAK mediating the expression of a variety of genes in response to cell stimuli, and thus plays a key role in many cellular processes such as cell growth and apoptosis. The activation of Stat 3 play a key role in CSCs in a variety of cancers and controls cell fate determination, survival, proliferation, and the maintenance of stem cells. Consequently its ectopic inhibition by drugs has been proposed as an approach to eliminate CSCs (Kim SY et al.). Stat 3 was found to be upregulated in HCT8 iCSCs as depicted in **Figure 4**.

Mucins, adhesion and signaling molecules

Mucins are O-linked glycoproteins that are anchored to the apical surface of epithelial cells in several organs. It physiological function resides in preventing the invasion of pathogens due its physical-chemical barrier properties. Furthermore, mucins are involved in cell signaling cascades like Wnt, Notch, and Hedgehog. Its aberrant overexpression is associated with tumor metastatic processes. Recent evidences demonstrated that mucins play a major role in the cellular transformation and signaling alteration in the epithelial mesenchymal transition (**EMT**) process and also in the enrichment and maintenance of cancer stem cell populations. Aberrant expression of mucins is directly implicated in the refraction to immunosurveilance and immunosuppressive induction around the tumours and probably in the protection of local tumor stem cells (Ponnusamy MP et al, Anandkumar A et al.). Both Mucins 1 and 4 are overexpressed in HCT8 iCSCs (**Figure 4**)

Wnt pathway

The canonical Wnt/β-catenin pathway acts as a **co-activator** of **transcription factors** by accumulation of β-catenin in the cytoplasm. The Wnt signaling cascade is critically important in stem cell biology, both in homeostatic maintenance of tissues and organs through their respective somatic stem cells and in the CSC/tumor initiating cell population. Abnormal activation of this cascade has been found aberrantly upregulated in a wide variety of human cancers. Furthermore, it has been widely proposed as essential cascade for the self-renewal of CSCs. Regarding the implication of the Wnt/β-catenin pathway in colon carcinoma, it is also known that this cascade is constitutively activated in CS colonosphere-derived cells (Kanwar SS et al.). We searched for the deregulation in key transducers of this cascade and found that beta-catenin and GSK3 alpha and beta were upregulated in HCT8 iCSCs (Figure 4).

Expression of stemness markers in HCT8 iCSCs

EpCAM

EpCAM (Epithelial cell adhesion molecule) is a transmembrane glycoprotein expressed in epithelia and overexpressed in most carcinomas. It is involved in cell adhesion, signaling, migration, proliferation, differentiation, and metastasis. Furthermore, it has the capacity of upregulate some cyclins and several transcriptions factor e.g. **c-Myc**. Its role in the **EMT** in tumors has been widely discussed, deserving as a robust CSCs marker in colon carcinomas (Reviewed by Markus Munz et al.). The HCT8 iCSCs derived from etoposide express large amounts of this adhesion molecule as compared to its parental cells **(Figure 4)**.

CD44 and CD133 (**Considerations**)

Without any doubt there is a controversy regarding CD44 and CD133 as distinctive markers for stemness. In our opinion both markers are questionable for a reliable phenotyping of CSCs, since wildtype tumors express large quantities of such closters. Regarding CD44, it is known that this CD has several variants which are distinctly expressed depending on the tissue type. Working on some variants of CD44 (purchased from Abcam), we note in the past that depending on the histological origin, some of them were confined in fact to stemness-like features. Formerly, the Abcam Company offered several variants which are now unfortunately discontinued. Concerning CD133 it is widely described, and somehow accepted, that this CD is not restricted to somatic stem cells or cancer stem cells, since CD133-negative cancer cells can also initiate tumors. The most cell lines and primary tumor cultures that we posses express in fact large quantities of this CD, and if we take into consideration that in tumoral masse the subset of CSCs in tumors is much lower than 2%, such intensive expression reveals a strong percentage of these fractions what is in contradiction to the already accepted proportion of CSCs within a tumor. Thus, the usefulness of these CDs as CSC

marker remains uncertain. Anyway, we decided include both markers in this exposition for further considerations in combination or with other putative stemness markers.

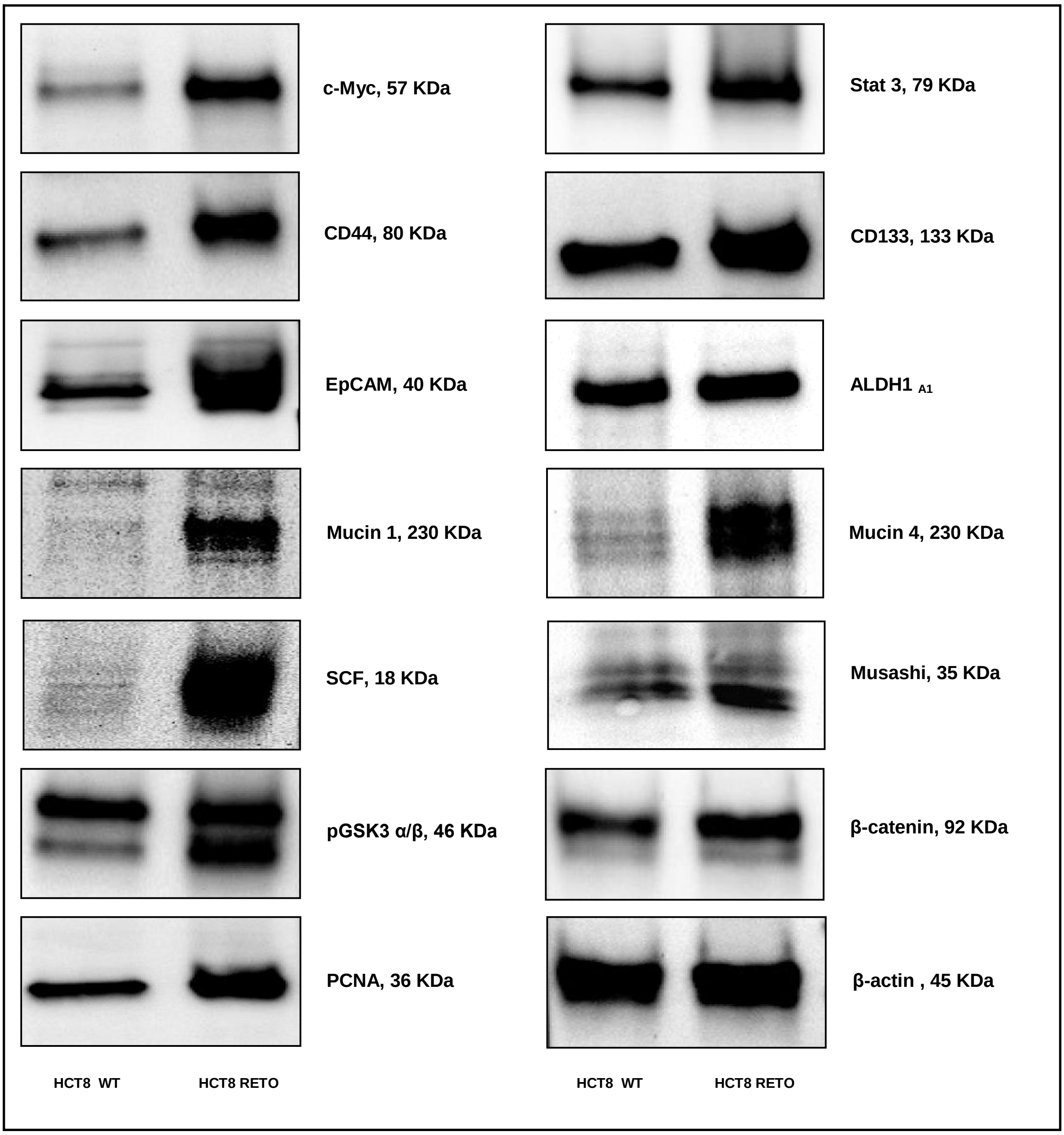

<u>Figure 4</u>: Set of different markers which are related to CSCs-features in colon carcinomas. The relative molecular weights are provided. Experiments are performed at least n = 3.

Simultaneous incubation of HCT8 RETO with amantadine and cisplatin, doxorubicin and 5FU on the IC_{50} values calculated for the drugs applied in combination for 24 hours is depicted in **Figure 5.**

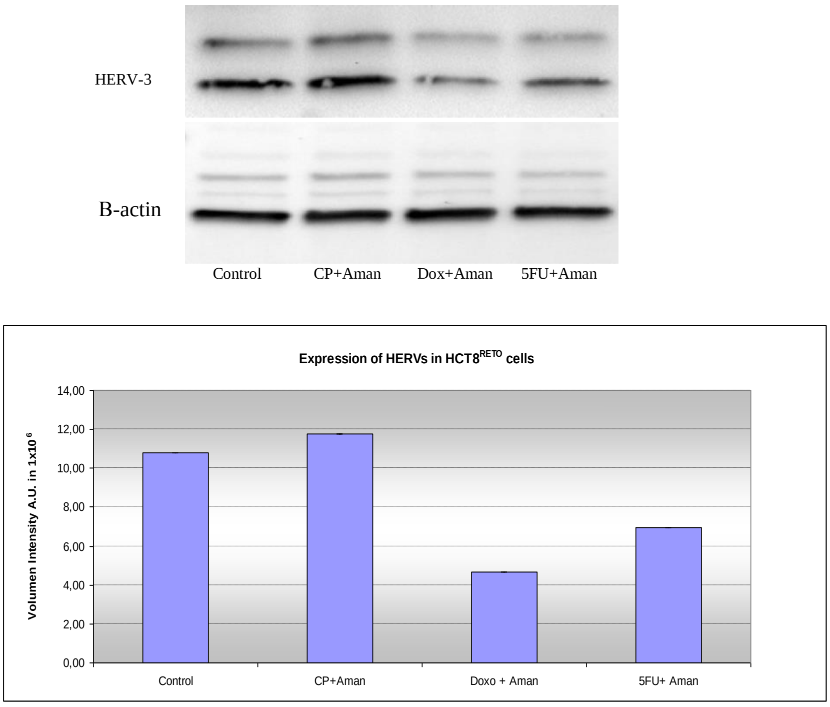

Figure 5: HERV-3 protein downregulation after antiviral exposure as analyzed by Western blot. The co-incubation of amantadine and cytostatics repress the expression of HERV proteins in HCT8[RETO] colon carcinoma cells (2x)

CP: Cisplatin

Aman: Amantadine

Doxo: Doxorubicin

5FU: 5-Fluorouracil

Literature

Anandkumar A and Devaraj H. Tumor Immunomodulation: Mucins in resistance to initiation and maturation of immune response against tumors. Scand J Immunol. 78(1), 2013.

Appalaraju Jaggupilli et al.. Significance of CD44 and CD24 as Cancer Stem Cell Markers: An Enduring Ambiguity. Clinical and Developmental Immunology. Volume 2012, Article ID 708036

B. Dave et al.. Inhibition of Breast Cancer Stem Cells (CSCs) by Novel STAT3 Inhibitors. Cancer Res, 69(24), 2009.

Denis N et al.. Stimulation of methotrexate resistance and dihydrofolate reductase gene amplification by c-myc. Oncogene, 6(8), 1991

Díaz-Carballo et al.. Atypical cell populations associated with acquired resistance to cytostatics and cancer stem cell features: the role of mitochondria in nuclear encapsulation. DNA Cell Biol, 33(11), 2014.

Kanwar SS et al.. The Wnt/beta-catenin pathway regulates growth and maintenance of colonospheres. Mol Cancer, 6;9:212, 2010.

Kim SY et al.. Role of the IL-6-JAK1-STAT3-Oct-4 pathway in the conversion of non-stem cancer cells into cancer stem-like cells. Cell Signal, 25(4), 2013.

Markus Munz et al.. The Emerging Role of EpCAM in Cancer and Stem Cell Signaling. Cancer Res, 69: (14), 2009.

Ponnusamy MP et al..Emerging Role of Mucins in Epithelial to Mesenchymal Transition. Curr Cancer Drug Targets. 2013 Nov;13(9):945-56.

Stephen P. Thomson and Frank L. Meyskens. Method for Measurement of Self-Renewal Capacity of Clonogenic Cells from Biopsies of Metastatic Human Malignant Melanoma. Cancer Research, 42. 4606-4613, 1982.

Zuqin Nie et al.. c-Myc is a universal amplifier of expressed genes in lymphocytes and embryonic stem cells. Cell, Vol. 151, 2012.
